# 726. Risk factor analysis for cefepime and piperacillin/tazobactam resistant organisms

**DOI:** 10.1093/ofid/ofad500.787

**Published:** 2023-11-27

**Authors:** Tho H Pham, Jeffrey Barletta, Vanthida Huang

**Affiliations:** R. Ken Coit College of Pharamcy/Arizona Department of Health Services, Phoenix, Arizona; Midwestern University College of Pharmacy-Glendale Campus, Glendale, Arizona; Midwestern University, College of Pharmacy-Glendale Campus, Glendale, Arizona

## Abstract

**Background:**

Carbapenems (CAR) are first line therapy for patients with infections caused by multidrug resistant organisms. As the prevalence of antibiotic resistance is increasing, CAR use has become a common choice for empiric therapy. This increase in use is concerning as prior CAR exposure is a risk factor for CAR resistance. Identification of risk factors associated with resistance for other beta-lactam antibiotics could aid in the appropriate use of CAR therapy while minimizing overuse. The objective of this study was to identify risk factors for cefepime (FEP) and piperacillin/tazobactam (PTZ) resistance and which population required CAR as an empiric therapy.

**Methods:**

This is a retrospective cohort conducted at HonorHealth John C Lincoln Medical Center, Phoenix AZ between January 2020 to July 2021. The case patients will be those who had an infection which is resistant to FEP or PTZ, while control patients have infection which is susceptible to FEP or PTZ. Recurrence of infection after receiving appropriate antimicrobial therapy considered as a separate case. Patients who do not have antimicrobial susceptibilities were excluded. Variables were compared between groups. Multivariate analysis using logistic regression was used to control for confounding variables.

**Results:**

Overall, 230 patients met inclusion criteria and were evenly distributed between cohorts. The mean age of the population at baseline was 64 ± 17.4, 111 (48.3%) patients were in an ICU and 103 (44.8%) patients required mechanical ventilation. Clinical variables which were significant in univariate analysis were included in multivariate analyses. Multivariate analyses revealed prior resistance to FEP or PTZ in the past 12 months (OR 8.35, 95% CI [2.28-30.50], p=0.001), transferred from outside facility (OR 3.40, 95% CI [1.80-6.42], p< 0.001), prior ICU admission in the past 90 days (OR 4.40, 95% CI [1.72-11.27], p=0.002), and prior antibiotic use in the past 30 days (OR 2.60, 95% CI [1.33-5.09], p=0.005) were associated with increased risk of FEP- or PTZ-resistant infections.

**Conclusion:**

Patients with prior resistance to FEP and PTZ within the last 12 months can benefit by initiating empiric therapy with CAR due to the increased risk for subsequent FEP- or PTZ-resistant organisms. Further investigation is warranted.

**Disclosures:**

**All Authors**: No reported disclosures

